# Decomposing Racial and Ethnic Disparities in Risk and Protective Factors of Dementia in the U.S

**DOI:** 10.1080/07317115.2025.2534651

**Published:** 2025-07-17

**Authors:** Nasim B. Ferdows, María P. Aranda

**Affiliations:** a Department of Public Health and Health Sciences, Bouvé College of Health Sciences, Northeastern University, Boston, Massachusetts, USA;; b School of Public Policy and Urban Affairs, College of Social Sciences and Humanities, Northeastern University, Boston, Massachusetts, USA;; c USC Suzanne Dworak-Peck School of Social Work, University of Southern California, Los Angeles, CA, USA;; d Edward R. Roybal Institute on Aging, University of Southern California, Los Angeles, CA, USA

**Keywords:** Blinder-Oaxaca decomposition, cognitive functioning, lifespan, Racial/Ethnic disparities

## Abstract

**Objectives:**

This study investigates racial/ethnic disparities in dementia risk and protective factors using data from the Health and Retirement Study (HRS) and the Harmonized Cognitive Assessment Protocol (HCAP).

**Methods:**

A retrospective analysis of 3,495 individuals aged 65+ from the 2016 HCAP linked to the HRS was conducted. Cognitive status was assessed using the Mini-Mental State Examination (MMSE) scores. Risk factors included midlife cardiovascular conditions, hearing loss, current smoking, depression, and physical inactivity. Protective factors were education and wealth. The Oaxaca-Blinder decomposition method was used to quantify the contribution of these factors in explaining racial/ethnic disparities in cognitive functioning.

**Results:**

Black participants had 2.883 times higher odds of developing dementia compared to Whites, while Hispanic participants had 1.230 times higher odds (not statistically significant). Mid- and late-life risk and protective factors explained 32% of the cognitive gap between Black and White participants, and 70% between Hispanic and White participants, leaving 68% and 30% unexplained, respectively.

**Conclusions:**

Addressing disparities in education, wealth, cardiovascular risks, depression, and hearing loss can reduce cognitive dysfunction in older adults.

**Clinical implications:**

Clinicians should target modifiable risk factors like depression and physical inactivity, particularly in minority populations. Addressing socioeconomic disparities is also crucial for improving cognitive health.

Along with physical decline, a decline in cognitive functioning is associated with aging and is predictive of mortality. Declining cognitive functioning frequently results in functional impairment and disability (McGuire et al., 2006). Recent population-based studies suggest that improved population cardiovascular health and rising levels of education in the past 25 years were associated with a reduction of dementia risk in the US (Bancks et al., 2019). Although cognitive functioning among older adults in the US has improved over time (Bancks et al., 2019) and dementia incidence and prevalence has declined in the last couple of decades (Derby et al., 2017; Satizabal et al., 2016; Wolters et al., 2020), the number of Americans aged 65 and older with Alzheimer’s dementia is projected to increase from 6.2 million in 2021 to 12.7 million by 2050 (“2024 Alzheimer’s disease facts and figures,” 2024) The cost of care for Alzheimer’s and other dementias is projected to increase from $355 billion to $1.1 trillion over this same period (“2024Alzheimer’s disease facts and figures,” 2024).

Lifestyle factors play a crucial role in an individual’s risk of developing dementia (Livingston et al., 2017). While non-modifiable risk factors such as older age and lower levels of early education contribute to the risk (Bancks et al., 2019; Zhu et al., 2018), modifiable lifestyle factors, including cardiovascular disease-related factors (e.g., physical inactivity, diabetes, obesity, smoking, hypertension, and high cholesterol), as well as hearing loss and depression, have been identified as significant contributors to dementia risk (Bachman et al., 2003; Livingston et al., 2017, 2020; Sol et al., 2020). Furthermore, there are notable variations in the prevalence and management of chronic diseases and behaviors associated with poor cardiovascular health among different racial and ethnic groups. Racial/ethnic disparities in dementia prevalence have been well-documented, with studies consistently demonstrating a higher risk of dementia among Black and Hispanic individuals compared to Whites (Chen & Zissimopoulos, 2018; Manly et al., 2022; Mehta & Yeo, 2017; Rovner et al., 2018), and a narrowing disparities in neurodegenerative diseases incidence (Lusk et al., 2023).

Lower educational attainment has been identified as a potential driver of these disparities, particularly among racial and ethnic minorities. Educational disparities (Amariglio et al., 2020; McDougall et al., 2007; Shadlen et al., 2006) have been associated with an increased risk of dementia, with findings suggesting that the higher prevalence of dementia among racial and ethnic minorities may be partially attributed to lower education levels, particularly among Blacks and Hispanics (Rodriguez et al., 2018). According to another study, it was found that years of education had a significant impact on reducing the odds of cognitive impairment and dementia across different race/ethnicity groups (Garcia et al., 2018). Other factors such as age, income level, and neurocognitive function have also been implicated in racial and ethnic differences in dementia risk.

In this study, our original contribution is two-fold. First, we extend prior work by using a nationally representative and longitudinal dataset to quantify mid- and late-life risk and protective factors that are associated with dementia in later life. Second, we employ Oaxaca-Blinder decomposition to decompose racial/ethnic disparities in lifespan characteristics associated with cognitive performance. This approach provides a novel framework for quantifying the proportion of disparities explained by measurable factors versus those that remain unexplained, addressing structural and unmeasured influences on cognitive disparities. By investigating these factors, this study seeks to enhance our understanding of the complex relationships between risk factors, racial/ethnic disparities, and cognitive decline in older adults, with the goal of informing interventions and policies to promote brain health and reduce cognitive disparities in the population. Figure 1 presents a conceptual framework that illustrates how demographic, midlife, and late-life factors contribute to racial and ethnic disparities in cognitive functioning, as evaluated through Oaxaca-Blinder decomposition.

## Methods

### Data

This study utilized data from the 2016 Harmonized Cognitive Assessment Protocol (HCAP) (Weir et al., 2016), a cohort within the Health and Retirement Study (HRS). The HRS is a nationally representative, biennial, longitudinal survey focusing on individuals aged 51 years or older in the US. It collects information on various aspects such as health, cognition, disability, work, and economic status of Americans. The HRS is sponsored by the National Institute on Aging (grant number NIA U01AG009740) and is conducted by the University of Michigan (HRS, 2023). It provides a core resource for researchers who require US population-level data on risk factors, care, cost, and other outcomes associated with dementia. The protocol, methods, and consent procedures for the original study were approved by the University of Michigan Institutional Review Board (IRB).

The entry cohort of the HCAP data consisted of a random selection of 5500 individuals from the wave 13 of HRS panel in 2016, with 3496 completing HCAP interviews. HCAP provides in-depth data on participants based on their prior participation in HRS waves. Details of HCAP have been previously published (David et al., 2014; Jones et al., 2024; Langa et al., 2020). We also linked the data to the RAND-HRS dataset, which offers a clean and user-friendly version of the HRS data, including derived measures for total annual income, wealth, and other key variables.

### Study population

Linking HCAP to HRS, we traced 3496 HCAP participants back to 2000 (9 waves) to capture their midlife characteristics. We restricted our sample to non-Hispanic White, non-Hispanic Black, and Hispanic adults aged 65 and older. We excluded 76 respondents who identified as others, as well as 149 individuals with missing Mini-Mental State Examination (MMSE) score information. Our analytic sample included 3271 individuals (72.85% non-Hispanic Whites, 16.05% non-Hispanic Blacks, and 11.10% Hispanics).

### Dementia measures and outcomes

In this study, cognitive functioning was assessed using the Mini-Mental State Examination (MMSE) score, a widely recognized and the most famous global measure of cognitive function and dementia (Nichols et al., 2024). The MMSE, introduced in the HRS study within the HCAP subsample, consists of 22 questions assessing various cognitive domains: 10 questions on orientation, 8 on language, and one each on registration, memory, spelling backward, and construction. Scores ranges from 0 to 30, with higher scores indicating better cognitive functioning. Based on the HCAP test selection criteria, we applied established cut points from prior studies to classify cognitive status: scores above 25 indicate *healthy cognition*, scores of 18–24 indicate *mild cognitive impairment (MCI)*, and scores below 17 indicate *dementia* (David et al., 2014).

While the MMSE is a widely used and validated screening tool, it is not a comprehensive diagnostic assessment. Its use in this study provides a standardized approach to cognitive classification but may not capture the full complexity of cognitive functioning. MMSE scores were not adjusted or normalized based on education; however, all regression models included education as a covariate to account for its influence while preserving the clinical interpretability of the original MMSE score.

While the HRS primarily uses the modified personal communication for Cognitive Status (TICS) to assess cognition (Crimmins et al., 2011; Langa et al., 2017), this measure is operationalized through the Langa-Weir classification system. It has been noted that TICS-based measures may be particularly sensitive when applied to racial/ethnic minority populations, potentially leading to an over-identification of cognitive impairment and dementia in these groups (Gianattasio et al., 2019). In contrast, the MMSE is administered through in-person interviews in HCAP, facilitates cross-national comparisons and provides a more detailed assessment of cognitive functioning. To ensure validity, we compared MMSE-based classifications with the Langa-Weir (TICS-based) classifications (see Supplemental Table 1), finding consistent results across measures. This further supports the use of the MMSE as a robust cognitive assessment tool within the HRS population.

**Midlife characteristics** were derived from the HRS and included history of various cardiovascular risk factors, such as hypertension, stroke, heart disease, obesity, alcohol misuse, and uninsurance, from 2000 to 2014. Alcohol misuse was defined according to the National Institute on Alcohol Abuse and Alcoholism definitions using criteria for “heavy alcohol use” based on gender and frequency of consumption (NIAAA, 2022). History of alcohol misuse was identified for individuals who met the criteria for heavy drinking in any year from 2000 to 2014. Hearing loss was assessed through participant self-assessment and the use of a hearing aid, resulting in two measures: history of wearing a hearing aid and self-reported fair or poor hearing with or without a hearing aid from 2000 to 2014.

Late-life (current) characteristics included smoking status, household wealth, depression, and physical inactivity – all measured in the last wave of data. Smoking status was categorized as “never,” “former,” or “current smoker.” Household wealth was categorized as “less than 53 000,” “53 001–178 000,” “178 001–470 000,” and “more than 470 001” (Chen & Zissimopoulos, 2018).

Depressive symptomology in the HCAP cohort was evaluated using the Center for Epidemiological Studies Depression Scale (CESD). The CESD score is calculated by summing the responses to five negative indicators (reflecting symptoms such as feeling depressed, having difficulty with tasks, restless sleep, loneliness, and sadness) and subtracting the responses to two positive indicators (representing feelings of happiness and enjoyment of life). Scores on the CESD range from 0 to 10, with higher scores indicating a higher presence of depressive symptoms. To categorize individuals as depressed, we considered a score above 3 as the threshold (Stephanie & Herzog, 2004).

*Physical inactivity* was derived by combining measures of current physical activity (light, moderate, or vigorous) and exercise (every day, more than once per week, 1–3 times per month, or never). Combining the severity and frequency measures, we categorized an individual as inactive if the person reported having less than once a week of all 3 types of physical activities during a month (Hong et al., 2023; Wei et al. 2025).

Demographic variables included age groups, gender, race/ethnicity, and mutually exclusive indicators for non-Hispanic Whites, non-Hispanic Blacks, and Hispanics – referred to Whites, Blacks and Hispanics henceforth. We also included current insurance status: only Medicare, Medicare and Medicaid, Medicare and Private, and other or no insurance.

### Statistical analysis

**Figure 2.**
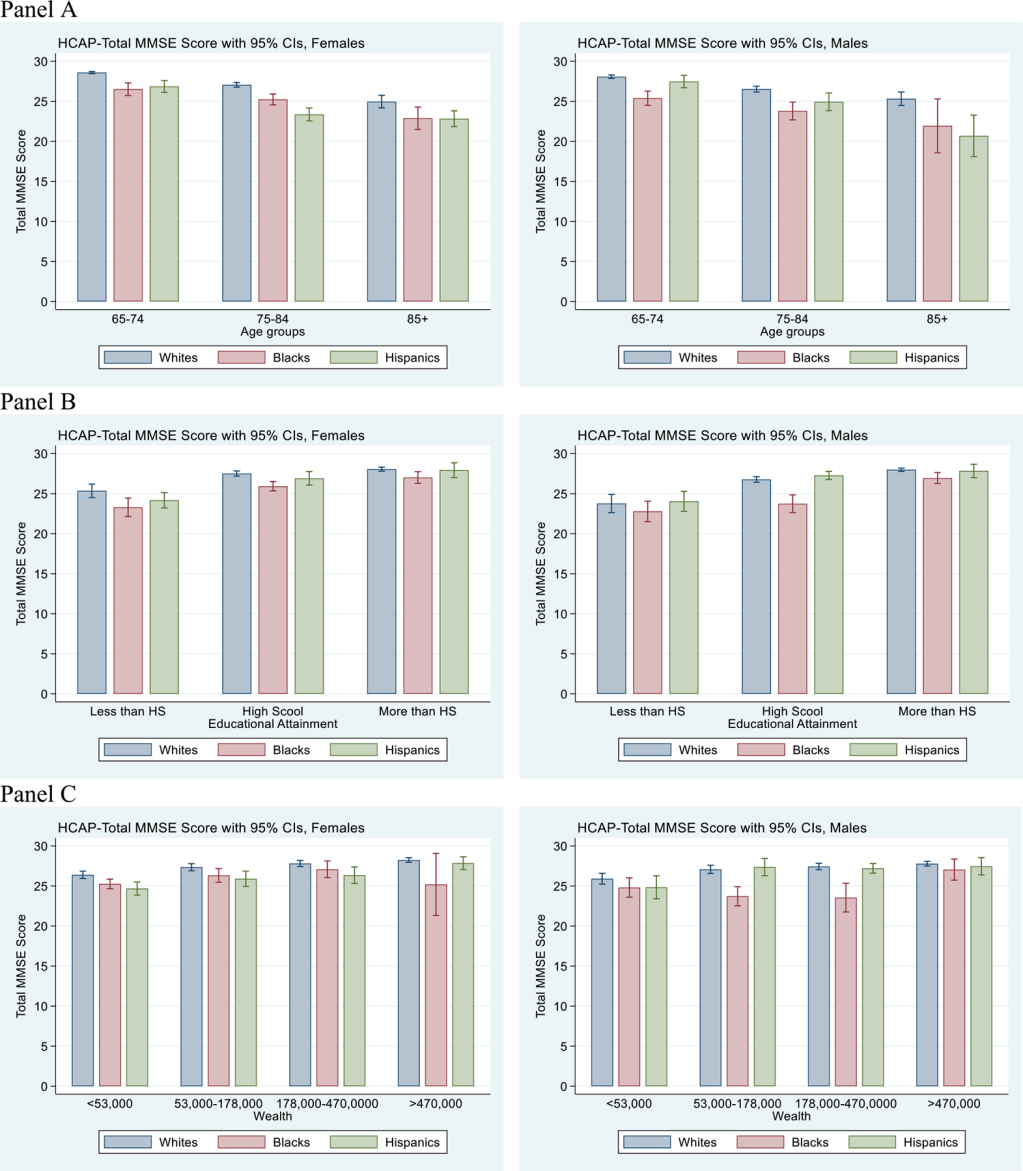
Racial/Ethnic difference in gender-specific MMSE scores of HCAP participants by socioeconomic categories, 2016. Abbreviations: MMSE, Mini-Mental State Examination; HCAP, Harmonized Cognitive Assessment Protocol; CI, confidence interval; HS, high school.

In descriptive analysis, we used HRS sampling weights and reported weighted percentages (Table 1). Sampling weights were applied to account for the complex survey design and to ensure that estimates are nationally representative. We further investigated gender-specific racial/ethnic differences associated with cognitive functioning scores, employing HRS sampling weights and reporting confidence intervals to identify statistically significant differences (Figure 2).

To examine the association between risk and protective factors of dementia across the lifespan, we conducted multivariable logistic regression analyses. The binary outcome was whether the individual has normal cognition and the reference group included those with dementia or mild cognitive impairment (MCI). These analyses accounted for the complex design of the HRS, incorporating appropriate sampling weights to ensure accurate inferences and generalizability to the non-institutionalized American population aged 65 and older. Four regression specifications were employed, progressively adding explanatory variables to the equation. The baseline regression included race/ethnicity indicators and sociodemographic variables (Model 1). The second specification included midlife characteristics (Model 2), and the third specification added late-life characteristics (Model 3). Additionally, we conducted a specification test that considered current insurance status while excluding the history of being uninsured, due to potential collinearity (Model 4) (Table 2).

To assess the extent to which observable characteristics explain variation in cognitive functioning scores, we performed Oaxaca-Blinder decomposition by racial/ethnic groups (Blinder, 1973; Jann, 2008; Oaxaca, 1973; Sinning et al., 2008). Originally developed to study Black – White wage gaps, the Oaxaca-Blinder decomposition partitions an observed mean difference in an outcome into two components. The “endowment” component (or “composition” effect) estimates how much of the gap would disappear if minoritized and reference groups had identical levels of measured characteristics (e.g., age, education, cardiovascular risk factors, depressive symptoms). The “coefficient” component (or “unexplained” portion) reflects the remaining difference attributable to unequal effects or returns to those characteristics (i.e., regression coefficients). This decomposition provides an advantage over conventional approaches – such as covariate-adjusted regression, marginal standardization, or interaction terms – which can estimate an overall disparity but do not distinguish how much is due to differences in group characteristics versus how those characteristics operate. A full algebraic presentation, including our counterfactual construction, is provided in Supplement A.

We implemented Oaxaca-Blinder using stratified linear regression by racial/ethnic category to disaggregate differences in cognitive functioning scores. By applying one racial/ethnic group’s characteristics to coefficients estimated using a different racial/ethnic group’s subsample, we quantified the extent to which differences in characteristics contribute to the cognitive functioning score disparity. The unexplained portion is used as a measure of disparity, but it is important to recognize that it also captures all the potential effects of differences in unobserved variables. Oaxaca-Blinder decomposition is a widely used approach in studying disparities, including racial/ethnic and rural/urban disparities (Angrisani et al., 2021; Spencer et al., 2018).

## RESULTS

### Sample description

Table 1 presents the characteristics of the HCAP cohort stratified by race/ethnicity. White participants had a slightly higher mean age, a lower proportion of females, and higher education levels. Black participants exhibited higher midlife prevalence rates of hypertension, stroke, and obesity, whereas Whites had a higher prevalence of heart disease. Hispanic participants demonstrated higher rates of poor hearing, lack of hearing aid use, and uninsured status. Current risk factors revealed higher smoking rates and physical inactivity among Black individuals, and a higher prevalence of depression among Hispanic individuals.

Panels A, B, and C illustrate the contributions of age, education, and wealth to cognitive functioning disparities across racial/ethnic groups by gender. Panel A shows that cognitive functioning declines with age across all groups, with White participants consistently scoring higher than Black and Hispanic participants. Panel B highlights the positive association between education and cognitive scores, with greater educational attainment mitigating disparities but not fully closing the gap. Panel C demonstrates that higher wealth is associated with better cognitive functioning, yet significant racial/ethnic differences persist even among those in higher wealth categories. Together, these panels underscore that while age, education, and wealth are important contributors to cognitive functioning, racial/ethnic disparities remain evident across all levels of these factors.

### Risk and protective factors for dementia

Column 1 of Table 2 shows that after accounting for sociodemographic characteristics, Black and Hispanic participants had higher odds of dementia compared to White participants (3.6, *p* < .01 and 1.547, *p* < .05, respectively). Having a high school education or more was associated with lower odds of dementia compared to individuals with no high school education (odds ratios of 0.3 and 0.1, respectively). When considering midlife characteristics, a history of stroke and alcohol misuse were associated with higher odds of developing dementia. Surprisingly, individuals wearing a hearing aid who had experienced hearing loss in midlife also had increased odds of dementia. Considering late-life characteristics, both depression and physical inactivity were associated with increased odds of dementia. Notably, physical inactivity had the most prominent effects among all lifespan factors examined.

### Racial/Ethnic disparities in dementia

Table 3 presents the results of the Oaxaca-Blinder decomposition, analyzing the mean predictions, observed gap, and the decomposition of cognitive functioning scores by race/ethnicity. White participants had a mean cognitive functioning score that was 2.1 points higher than Black participants. Around 32% of this difference was explained by demographics and late-life characteristics. These characteristics are grouped under the endowments term, which shows that if Black participants had the same demographic, midlife, and late-life characteristics as White participants, their cognitive functioning score would increase by 0.6 points. This reflects the portion of the cognitive gap attributable to measured characteristics. The remaining 68% of the gap is unexplained, indicating differences that cannot be attributed to measured characteristics. The coefficients term indicated that if Black participants had the same returns (or effects of characteristics) as those observed in White participants, their cognitive functioning scores would increase by 1.4 points. The interaction term, which captures the combined effect of differences in characteristics and returns, was not found to be significant, indicating that simultaneous differences in these two dimensions did not contribute meaningfully to the gap.

When comparing White and Hispanic participants, the mean cognitive functioning score for White participants was 1.8 points higher than for Hispanic participants. Demographics and late-life characteristics accounted for approximately 70% of this difference, while about 30% remained unexplained. The increase of 1.3 points suggested that differences in demographics, midlife, and late-life characteristics explained around 70% of the cognitive functioning difference. The coefficients term indicated that if Hispanic participants had the same demographic, midlife, and late-life characteristics as those observed in White participants, their cognitive functioning scores would increase by 0.6 points.

### Sensitivity analysis

To validate the use of the MMSE score as a continuous measure in the Oaxaca-Blinder decomposition, we conducted multivariate ordinary least squares regression using sampling weights. The results were consistent with the previous findings using the discrete outcome measure, demonstrating the robustness and comparability of the results (Supplemental Table 2).

We initially performed a sensitivity analysis to explore the potential impact of traumatic brain injuries (TBI) as a risk factor for dementia. However, due to the limited sample size, with only 224 individuals completing the TBI questionnaire out of the matched HCAP respondents, and insufficient statistical power, this analysis was not included in the final manuscript.

Furthermore, we compared Hispanic and Black participants and performed an Oaxaca-Blinder decomposition analysis. When comparing cognitive functioning between Black and Hispanic participants (Supplemental Table 3), no statistically significant differences in mean cognitive scores were observed (mean difference: −0.32, *p* = .37). The Oaxaca-Blinder decomposition showed that demographic characteristics accounted for 67% of the observed difference. However, a significant portion (−1.1 points) was attributed to unexplained differences in coefficients, indicating potential disparities in how characteristics impact cognitive outcomes across these groups.

## Discussion

Our study extends the existing literature on racial/ethnic disparities in cognitive functioning by applying Oaxaca-Blinder decomposition to quantify the contributions of measurable factors and unexplained influences. This approach provides a novel framework for addressing disparities and identifying opportunities for targeted interventions. Thus, this study is the first to investigate racial/ethnic disparities in dementia risk and protective factors throughout mid- and late-life, utilizing a large, nationally representative survey of Americans aged 65 and older. The study reveals four key findings: (1) Higher levels of education and wealth were associated with reduced odds of dementia. (2) Midlife factors such as stroke and hearing loss, as well as late-life factors including depression and physical inactivity, were linked to an increased likelihood of dementia. (3) After accounting for sociodemographic, midlife, and late-life characteristics, Black participants exhibited the highest odds of developing dementia, followed by Hispanic and White participants. (4) The Oaxaca-Blinder decomposition analysis revealed that approximately 68% of the disparity in cognitive functioning scores between Black and White participants remained unexplained, even after considering demographics and late-life factors. Additionally, when comparing White and Hispanic participants, nearly 30% of the disparity in their cognitive functioning scores remained unexplained after accounting for demographics and late-life characteristics.

### Risk and protective factors for dementia

Our analysis identified several risk and protective factors for dementia. Midlife cardiovascular risk factors, such as a history of stroke and hearing loss, along with late-life factors like depression and physical inactivity, were found to increase the odds of dementia. Interestingly, hearing aid use in midlife was associated with a higher likelihood of dementia. This finding is counterintuitive and may reflect reverse causality – where individuals with early signs of cognitive decline are more likely to use hearing aids – or unmeasured confounding by the severity of hearing loss. Future studies with more granular hearing data may be better suited to disentangle these relationships. Conversely, higher educational attainment and wealth were associated with a lower risk of dementia. These findings align with previous research highlighting the impact of cardiovascular health and lifestyle factors on dementia risk (Livingston et al., 2020; Whitmer et al., 2005, 2008). However, contrary to some prior studies, we did not observe a significant association between midlife obesity and dementia risk.

Regarding late-life characteristics, our results support previous findings that link depressive symptoms to a faster decline in memory (Livingston et al., 2017, 2020; Sol et al., 2020) and report a decreased risk of dementia with increased physical activity (Marioni et al., 2015). Furthermore, physical inactivity was associated with higher odds of dementia (Livingston et al., 2017, 2020). Although smoking has been linked to dementia in previous studies (Bachman et al., 2003; Livingston et al., 2017, 2020), we did not find a significant association, although current smokers did exhibit higher odds of dementia compared to former or never smokers.

### Racial/Ethnic disparities in cognitive functioning

Prior research consistently demonstrates that Black and Hispanic older adults have a higher prevalence, incidence, and risk of dementia compared to White older adults Chen and Zissimopoulos (2018); Farina et al. (2020); Manly et al. (2022); Rovner et al. (2018); Weuve et al. (2018). Even after accounting for sociodemographic, risk, and protective factors, racial/ethnic disparities in dementia persist Chen and Zissimopoulos (2018); Farina et al. (2020); Sol et al. (2020); Weuve et al. (2018). Most studies report that lower educational attainment (Amariglio et al., 2020; Garcia et al., 2018) and higher risk factors (Levine et al., 2020) among Black and Hispanic populations contribute to lower cognitive function and higher dementia rates compared to Whites. However, 68% of the disparity between Black and White participants and 30% of the disparity between Hispanic and White participants remained unexplained. Unexplained differences in cognitive functioning among Black and Hispanic participants compared to Whites may be attributed to structural racism, perceived discrimination, and healthcare stereotypes. Other factors such as pollution, environmental/chemical exposures, and biological factors may also contribute to these disparities. The large unexplained gap, particularly between Black and White participants, underscores the limitations of individual-level predictors in fully capturing systemic disadvantage. Future studies should incorporate more direct measures of structural and social determinants of health to better understand and address these persistent disparities. Measurement error, particularly in self-reported measures and the cognitive test used to assess dementia, poses limitations in our study. While self-reported health is generally reliable, the accuracy of self-reported disease incidence and functioning may vary. Additionally, the complex relationships between educational attainment, midlife factors, and cognitive functioning could not be fully explored due to the nature of the Oaxaca-Blinder decomposition method used. Furthermore, the dementia measure used in this study is based on a cognitive test and is therefore subject to measurement error. Measurement error may vary across racial/ethnic groups and may be more notable for Blacks and Hispanics if correlated with factors such as educational attainment (Figure 2). While the MMSE is widely used and validated as a cognitive screening tool, it is not a comprehensive diagnostic assessment. It may exhibit differential measurement properties across racially and educationally diverse populations, leading to potential under- or over-estimation of cognitive impairment. This variability may contribute to the unexplained portion of disparities observed in our decomposition analysis. Although we controlled for education and other relevant factors, the potential influence of measurement error should be considered when interpreting these findings.

While we present gender-stratified results, our study was not specifically designed to examine the intersection of race and gender. Intersectional analyses that explore how race, gender, and other social identities interact to influence cognitive aging are crucial for capturing the full complexity of disparities. Future research using larger or pooled samples may help illuminate how these intersecting factors jointly shape cognitive health outcomes.

The comparison between Black and Hispanic participants highlights unique patterns in cognitive disparities. While demographic characteristics, such as age and education, partially explain differences, the unexplained portion underscores the potential role of cultural, environmental, or unmeasured factors in shaping cognitive outcomes. These findings suggest that interventions targeting social determinants of health may be particularly effective in addressing disparities within and across minority groups.

This study builds upon earlier preliminary findings presented at the Gerontological Society of America (Ferdows & Aranda, 2022), where initial results were shared as part of a scientific abstract. The current manuscript significantly expands on those findings by employing decomposition analyses to quantify disparities and integrating additional data and methodological rigor to address these disparities.

## Conclusions

Declining cognitive functioning in late life results in functional impairment and disability. Our analysis indicates that differences in sociodemographic, midlife, and late-life risk and protective factors for dementia explained a substantial proportion of the difference in cognitive functioning of older adults of different racial/ethnic groups. These findings provide information for prioritizing the development of public health interventions and resource allocations to optimize the cognitive health of all racial/ethnic groups of future generations. The information can inform the development of targeted interventions, health promotion strategies, and policies aimed at reducing dementia risk and improving cognitive health outcomes, particularly among marginalized populations.

## Supplementary Material

Supp 1

Supplemental data for this article can be accessed online at https://doi.org/10.1080/07317115.2025.2534651

## Figures and Tables

**Figure 1.**
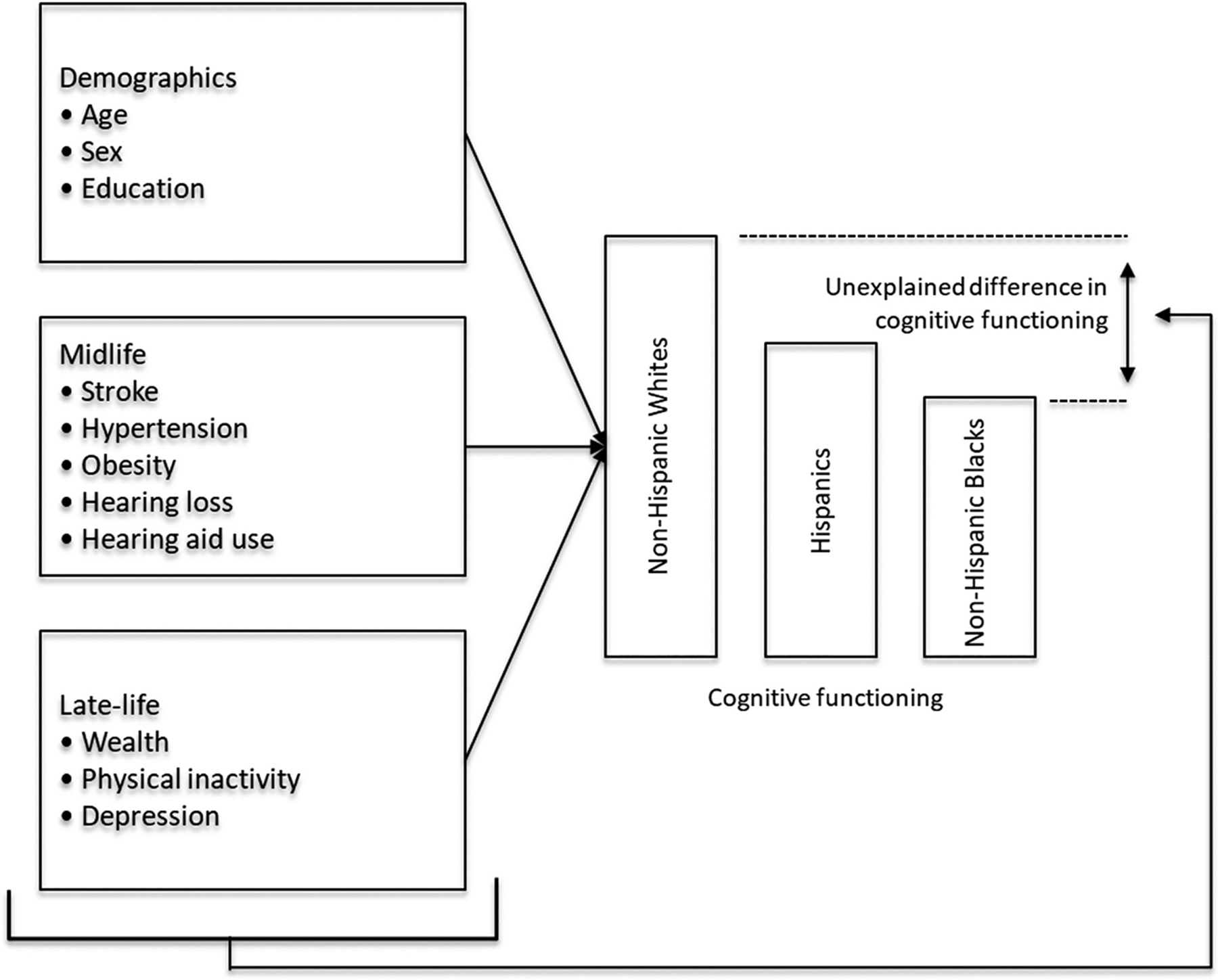
Conceptual framework for decomposing racial and ethnic disparities in cognitive functioning using life course factors.

**Table 1.** Characteristics of the 2016 HCAP participants by race/ethnicity.

Characteristics	Non-Hispanic Whites		Non-Hispanic Blacks		Hispanics		Total	
	(*n* = 2383)		(*n* = 525)		(*n* = 363)		(*n* = 3271	
	%^a^%	SE	%^a^	SE	%^a^	SE	%^a^	SE
**MCI/Dementia** ^b^	9.6%	0.5	28.2%	2.8	24.3%	2.9	12.4%	0.6
**Demographic characteristics**								
Age (years)	74.4	0.2	73.5	0.6	73.3	0.5	74.2	0.2
Females	55.3%	1.3	66.0%	2.6	61.2%	4.1	56.7%	1.1
**Early life characteristics**								
Education								
Less than high school	8.5%	0.7	25.0%	3.2	51.0%	4.0	13.2%	0.9
High school	33.7%	1.2	35.2%	2.4	22.6%	3.1	33.0%	1.0
More than high school	57.9%	1.5	39.8%	2.5	26.4%	3.0	53.8%	1.4
**Midlife characteristics**								
Cardiovascular risk factors								
Hypertension	61.6%	1.3	82.1%	2.3	65.3%	5.8	63.8%	1.2
Stroke	8.3%	0.7	12.7%	2.5	7.2%	1.7	8.6%	0.7
Heart disease	28.5%	0.9	26.9%	3.1	21.7%	3.8	27.9%	0.9
Obesity	46.0%	1.4	51.9%	3.2	50.1%	3.1	46.8%	1.2
Alcohol	16.4%	0.9	9.4%	1.7	14.2%	2.7	15.6%	0.8
Hearing loss								
Good hearing	63.1%	1.3	59.6%	3.5	40.9%	4.0	61.1%	1.3
Poor hearing, wearing aid	13.6%	0.9	5.4%	1.1	11.3%	1.8	12.7%	0.8
Poor hearing, not wearing aid	23.2%	1.0	35.0%	3.5	47.8%	3.4	26.2%	1.0
History of no insurance	13.1%	0.8	26.3%	3.2	38.8%	4.8	16.3%	1.0
**Late-life (Current) characteristics**								
Smoking								
Current	7.2%	0.8	11.5%	2.8	11.5%	2.3	8.0%	0.7
Former	48.4%	1.1	50.2%	3.1	41.3%	4.9	48.0%	1.0
Never	44.4%	1.1	38.3%	3.1	47.2%	4.8	44.0%	1.0
Depression	10.6%	0.7	17.8	2.0	22.4%	3.4	12.1%	0.6
Physical inactivity	12.4%	0.8	19.3%	2.3	13.6%	2.3	13.1%	0.7
Wealth								
< 53 000	17.8%	1.2	53.8%	3.2	48.6%	3.1	23.4%	1.2
53 001–178 000	18.6%	1.0	19.6%	2.0	23.7%	4.5	19.1%	0.9
178 001–470 000	23.0%	1.1	16.8%	2.7	17.3%	3.6	22.0%	1.1
470 001 +	40.6%	1.7	9.8%	1.7	10.4%	2.7	35.5%	1.7
Current insurance								
Medicare	69.7%	1.4	60.7%	3.2	51.0%	3.4	67.4%	1.3
Medicare & Medicaid	4.1%	0.5	15.7%	2.8	24.9%	3.6	6.7%	0.7
Medicare & private	21.2%	1.1	17.4%	2.9	10.8%	2.2	20.1%	1.0
Other or no insurance	5.1%	0.7	6.2%	1.3	13.3%	3.6	5.8%	0.7

Abbreviations: HCAP, Harmonized Cognitive Assessment Protocol; SE, standard error.

a Weighted percentages were derived using the HRS sampling weights to adjust for the complex design of the HRS survey.

b The MMSE score cut points is a score above 25 for healthy cognition, score of 18–24 for mild cognitive impairment(MCI), and score below 17 for dementia.

**Table 2.** Risk and protective factors of MCI/Dementia^a^ at age 65 years or older in the 2016 HCAP cohort.

Variables	Model 1 (OR)	Model 2 (OR)	Model 3 (OR)	Model 4 (OR)
**Demographics**				
Race/Ethnicity (ref.: Whites)				
Blacks	3.6*** (0.6)	3.4*** (0.6)	2.9*** (0.5)	2.9*** (0.6)
Hispanics	1.5** (0.3)	1.4* (0.3)	1.2 (0.3)	1.2 (0.2)
Age category (ref.: 65–74 years)				
75–84	3.5*** (0.5)	3.5*** (0.5)	3.3*** (0.6)	3.2*** (0.5)
85 +	7.9*** (1.7)	7.6*** (1.7)	5.7*** (1.4)	5.2*** (1.3)
Females	0.6*** (0.1)	0.7*** (0.1)	0.5*** (0.1)	0.5*** (0.1)
**Early life**				
Education (ref.: Less than high school)				
High school	0.3*** (0.0)	0.3*** (0.1)	0.3*** (0.1)	0.3*** (0.1)
More than high school	0.1*** (0.0)	0.2*** (0.0)	0.1*** (0.0)	0.2*** (0.0)
**Midlife**				
Cardiovascular risk factors				
Hypertension		1.0 (0.2)	0.9 (0.2)	0.9 (0.1)
Stroke		1.8*** (0.3)	1.6** (0.3)	1.5** (0.3)
Obesity		1.0 (0.1)	0.9 (0.1)	0.9 (0.1)
Alcohol		1.2 (0.2)	1.4 (0.3)	1.4* (0.3)
Hearing loss (ref.: Good hearing)				
Poor hearing, wearing aid		1.7*** (0.3)	1.6** (0.3)	1.5** (0.3)
Poor hearing, not wearing aid		1.7*** (0.3)	1.5** (0.3)	1.4* (0.3)
History of no insurance		1.3 (0.2)	1.3 (0.3)	
**Late-life (Current)**				
Smoking (ref.: Current)				
Former			1.1 (0.3)	1.1 (0.3)
Never			1.4 (0.5)	1.5 (0.5)
Depression			1.4** (0.2)	1.4* (0.2)
Physical inactivity			2.3*** (0.4)	2.3*** (0.4)
Wealth (ref. < 53 000)				
53001–178000			0.6*** (0.1)	0.6*** (0.1)
178 001–470 000			0.5****** (0.1)	0.6*** (0.1)
470 001 +			0.5** (0.1)	0.5** (0.1)
Current insurance (ref.: Medicare)				
Medicare & Medicaid				1.2 (0.3)
Medicare & private				0.7* (0.2)
Other or no insurance				1.3 (0.4)
Constant	0.3*** (0.1)	0.2*** (0.0)	0.2*** (0.1)	0.2*** (0.1)
Observations	3351	3338	3233	3218

Abbreviations. OR, odds ratio.

*Note*. Standard errors in parentheses.

*** *p* < .01,

** *p* < .05,

* *p* < .10.

a MMSE score cut points is a score above 25 for health cognition, score of 18–24 for mild cognitive impairment (MCI), and score below 17 for dementia.

**Table 3.** Racial and ethnic disparities in cognitive functioning at age 65 years or older in the 2016 HCAP cohort.

	Whites vs Blacks (n = 3023)		Whites vs Hispanics (n = 2837)	
	Whites	Blacks	Whites	Hispanics
Mean	27.7*** (0.1)	25.6*** (0.2)	27.7*** (0.1)	25.9*** (0.3)
Difference	2.1*** (0.2)		1.8*** (0.3)	
**Explained**				
Total	0.7*** (0.1)		1.3*** (0.2)	
Percent	32%		70%	
Demographics characteristics	0.3** (0.1)		0.8*** (0.1)	
Mid-life characteristics	0.0 (0.0)		0.1 (0.1)	
Late-life characteristics	0.4*** (0.1)		0.4*** (0.1)	
**Unexplained**				
Total	1.4*** (0.2)		0.5** (0.2)	
Percent	68%		30%	
**Decomposition**				
Endowments	0.6 (0.4)		1.3*** (0.3)	
Coefficients	1.4*** (0.2)		0.6** (0.2)	
Interaction	0.1 (0.4)		−0.1 (0.3)	

Standard errors in parentheses. Controlled for demographics (age categories, gender, and education), midlife factors (hypertension, stroke, obesity, alcohol, hearing loss, and history of not being insured during midlife), and current factors (smoking, depression, physical inactivity, and wealth).

*** *p* < .01,

** *p* < .05,

* *p* < .10

## Data Availability

The data that support the findings of this study are available from the University of Michigan at https://hrs.isr.umich.edu. Collection of these data were supported by the National Institute on Aging (NIA U01AG009740) and the Social Security Administration.

## Clinical Implications

- Prioritize prevention strategies for older Black and Hispanic adults by addressing modifiable risk factors such as depression, cardiovascular conditions, and physical inactivity.
- Promote education and address socioeconomic disparities to reduce dementia risks, particularly in minority populations.
- Recognize that a substantial portion of dementia risk remains unexplained, highlighting the role of structural and social determinants of health.
