# Supplementary material for: Decomposing Racial and Ethnic Disparities in Risk and Protective Factors of Dementia in the U.S
Source: Clin Gerontol. Author manuscript; Available in PMC 2025 Aug 13. (PMC12349561; doi:10.1080/07317115.2025.2534651)
Supplement: Supp 1 [file NIHMS2102328-supplement-Supp_1.docx]

# Supplementary Materials

***Supplement A. Racial and ethnic decomposition of cognitive functioning and dementia***

Using Oaxaca-Blinder decomposition, we compared each pair of racial/ethnic groups (Whites vs Blacks, Whites vs Hispanics and Blacks vs Hispanics). Here we only describe the methods comparing Whites and Blacks. Our regression models were specified as follows, for each pair:

$M_{i}=\beta_{0}+\beta_{1}{demographic}_{i}+\beta_{2}{early}_{i}+\beta_{3}{mid}_{i}+\beta_{4}{late}_{i}+\varepsilon_{i}$ (3)

where $M_{i}$ denotes Mini-Mental State Examination (MMSE) score. For simplicity, we represent all measured characteristics as $X$ and their coefficients as $\beta$:

$M=\beta_{0}+\beta X+\varepsilon$ (3)

We ran identically specified regressions for White, Black and Hispanic older adults, denoted by w, b, and h, respectively:

$M_{l}=X_{l}^{'}\beta_{l}+\varepsilon_{l} ; {E(\varepsilon}_{l})=0 \& l\in(w, b, h)$ (4)

Using the estimated coefficients from the equation for Black older adults’ equation ($\bar{\beta}_{b}$) and characteristics from the White older adult’s equation ($\bar{X}_{w})$, we generated counterfactual estimates as follows:

$$\hat{M}_{b}=\bar{X}_{w}\hat{\beta}_{b}$$

where $\hat{M}_{b}$ represents the expected MMSE score for Black older adults given average White older adults characteristics. The difference between the average observed Black older adults MMSE ($\bar{X}_{b}\hat{\beta}_{b}$) and the counterfactual estimate ($\bar{X}_{w}\hat{\beta}_{b}$) represents the explained variation in MMSE score, while the remainder of the differences is due to differences in coefficients of older Black and White adults, or differences in the marginal effect of individual characteristics on MMSE score, dubbed “unexplained” variation.

$$\Delta M=E\left( M_{b} \right)-E\left( M_{w} \right)=\bar{X}_{b}\beta_{b}- \bar{X}_{w}\beta_{w}=\bar{X}_{b}\beta_{b}- \bar{X}_{w}\beta_{w}+\bar{X}_{w}\beta_{b}- \bar{X}_{w}\beta_{b}$$

$=\left( \bar{X}_{b}-\bar{X}_{w} \right)\beta_{b}+\bar{X}_{w}\times\left( \beta_{b}-\beta_{w} \right)=\Delta M_{Explained}+\Delta M_{Unemplained}$

We repeated this estimation for each pair of racial/ethnic groups.

Supplemental Table 1. Cognitive functioning at age 65 years or older in the 2016 HCAP cohort—comparing HRS and HCAP measures

| **Cognitive functioning** | | Whites | | Blacks | | Hispanics | | Total | |
| --- | --- | --- | --- | --- | --- | --- | --- | --- | --- |
|  |  | %^a^ | SE | % ^a^ | SE | % ^a^ | SE | % ^a^ | SE |
| **Definition 1—MMSE** | |  |  |  |  |  |  |  |  |
|  | Demented | 2.07% | 0.3 | 5.51% | 1.2 | 5.27% | 1.2 | 2.65% | 0.3 |
|  | MCI | 8.99% | 0.6 | 24.41% | 2.7 | 20.10% | 2.8 | 11.31% | 0.6 |
|  | Healthy | 88.94% | 0.6 | 70.08% | 2.9 | 74.63% | 3.2 | 86.04% | 0.6 |
| **Definition 2— Langa–Weir** | |  |  |  |  |  |  |  |  |
|  | Demented | 3.81% | 0.4 | 13.31% | 1.6 | 15.34% | 2.7 | 5.62% | 0.5 |
|  | CIND | 15.03% | 0.7 | 34.65% | 3.7 | 25.70% | 2.5 | 17.70% | 0.9 |
|  | Normal | 81.17% | 0.8 | 52.05% | 3.8 | 58.96% | 3.5 | 76.68% | 0.9 |

*Abbreviations*. HCAP, Harmonized Cognitive Assessment Protocol; HRS, Health and Retirement Study; SE, standard error; MMSE, Mini-Mental State Examination; MCI, mild cognitive impairment; CIND, cognitive impairment no dementia.

*Note*. N = 3347.

The MMSE score cut points is a score above 25 for healthy cognition, score of 18-24 for mild cognitive impairment, and score below 17 for dementia.

HRS dementia for self-respondents is a score 6 or less, 7–11 for CIND, and 12+ (of 27) for normal. With proxy reports of mental status, instrumental activities of daily living difficulties, and interviewer assessment of cognition for proxy cases, a score 6+ (of 11) is demented, 3–5 is CIND, 2 or less is normal. HRS imputed these measures when missing, because they tend to be missing for the more cognitively impaired.

^a^Weighted percentages were derived using the HRS sampling weights to adjust for the complex design of the HRS survey.

Supplemental Table 2. Risk and protective factors of cognitive functioning at age 65 years or older in the 2016 HCAP cohort—using MMSE score as a continuous outcome

| **VARIABLES** | | **Model 1** | **Model 2** | **Model 3** | **Model 4** |
| --- | --- | --- | --- | --- | --- |
| **Demographics** | |  |  |  |  |
| Race/Ethnicity (ref.: Whites) | |  |  |  |  |
|  | Blacks | -1.789*** | -1.697*** | -1.401*** | -1.386*** |
|  |  | (0.212) | (0.220) | (0.208) | (0.206) |
|  | Hispanics | -0.699*** | -0.648** | -0.513** | -0.473** |
|  |  | (0.258) | (0.243) | (0.204) | (0.209) |
| Age category (ref.: 65-74 years) | |  |  |  |  |
|  | 75-84 | -1.378*** | -1.317*** | -1.019*** | -1.001*** |
|  |  | (0.134) | (0.140) | (0.110) | (0.107) |
|  | 85 + | -3.058*** | -2.838*** | -2.048*** | -2.028*** |
|  |  | (0.302) | (0.289) | (0.230) | (0.225) |
| Females | | 0.502*** | 0.324** | 0.518*** | 0.517*** |
|  |  | (0.130) | (0.144) | (0.126) | (0.125) |
| Education (ref.: Less than high school) | |  |  |  |  |
|  | High school | 2.221*** | 2.075*** | 1.750*** | 1.720*** |
|  |  | (0.214) | (0.211) | (0.180) | (0.191) |
|  | More than high school | 2.928*** | 2.674*** | 2.361*** | 2.323*** |
|  |  | (0.205) | (0.210) | (0.184) | (0.191) |
| **Midlife** | |  |  |  |  |
| Cardiovascular risk factors | |  |  |  |  |
|  | Hypertension |  | -0.109 | 0.0500 | 0.0466 |
|  |  |  | (0.143) | (0.116) | (0.117) |
|  | Stroke |  | -0.955*** | -0.669*** | -0.660*** |
|  |  |  | (0.237) | (0.209) | (0.208) |
|  | Obesity |  | -0.00977 | 0.0455 | 0.0558 |
|  |  |  | (0.102) | (0.0941) | (0.0925) |
|  | Alcohol |  | 0.0571 | -0.0427 | -0.0552 |
|  |  |  | (0.171) | (0.139) | (0.139) |
| Hearing loss (ref.: Good hearing) | |  |  |  |  |
|  | Poor hearing, wearing aid |  | -0.717*** | -0.520*** | -0.505*** |
|  |  |  | (0.220) | (0.152) | (0.153) |
|  | Poor hearing, not wearing aid |  | -0.708*** | -0.486*** | -0.480*** |
|  |  |  | (0.167) | (0.147) | (0.150) |
| History of no insurance | |  | -0.128 | -0.00285 |  |
|  |  |  | (0.131) | (0.152) |  |
| **Later Life (Current)** | |  |  |  |  |
| Smoking (ref.: Current) | |  |  |  |  |
|  | Former |  |  | 0.206 | 0.208 |
|  |  |  |  | (0.201) | (0.201) |
|  | Never |  |  | 0.111 | 0.103 |
|  |  |  |  | (0.216) | (0.215) |
| Depression | |  |  | -0.477*** | -0.400** |
|  |  |  |  | (0.179) | (0.175) |
| Supplemental Table 2 (continued). Risk and protective factors of cognitive functioning at age 65 years or older in the 2016 HCAP cohort—using MMSE score as a continuous outcome | | | | | |
| **VARIABLES** | | **Model 1** | **Model 2** | **Model 3** | **Model 4** |
| Physical inactivity | |  |  | -1.202*** | -1.175*** |
|  |  |  |  | (0.198) | (0.192) |
| Wealth (ref. < 53 000) | |  |  |  |  |
|  | 53 001-178 000 |  |  | 0.702*** | 0.654*** |
|  |  |  |  | (0.162) | (0.155) |
|  | 178 001-470 000 |  |  | 0.795*** | 0.727*** |
|  |  |  |  | (0.149) | (0.131) |
|  | 470 001 + |  |  | 0.816*** | 0.756*** |
|  |  |  |  | (0.182) | (0.165) |
| Current insurance (ref.: Medicare) | |  |  |  |  |
|  | Medicare & Medicaid |  |  |  | -0.341 |
|  |  |  |  |  | (0.270) |
|  | Medicare & private |  |  |  | 0.0513 |
|  |  |  |  |  | (0.140) |
|  | Other or no insurance |  |  |  | -0.0760 |
|  |  |  |  |  | (0.256) |
| Constant | | 25.57*** | 26.25*** | 25.60*** | 25.67*** |
|  |  | (0.221) | (0.259) | (0.332) | (0.330) |
| Observations | | 3,351 | 3,338 | 3,233 | 3,218 |

*Abbreviations*. MMSE, Mini-Mental State Examination.

*Note*. Standard errors in parentheses.

*** *P* < .01, ** *P* < .05, * *P* < .10.

Supplemental Table 3. Racial and ethnic disparities in cognitive functioning at age 65 years or older in the 2016 HCAP cohort—comparing Blacks and Hispanics

|  | **Blacks vs Hispanics (N = 916)** | |
| --- | --- | --- |
|  | **Blacks** | **Hispanics** |
| Mean | 25.57*** | 25.89*** |
|  | (0.236) | (0.268) |
| Gap | -0.316 (0.373) | |
| **Explained** |  | |
| Total | 0.683** (0.321) | |
| Demographic characteristics | 0.674*** (0.22) | |
| Mid-life characteristics | 0.00532 (0.103) | |
| Late-life characteristics | 0.00825 (0.0946) | |
| **Unexplained** |  | |
| Total | -0.999*** (0.278) | |
| **Decomposition** |  | |
| Endowments | 0.421 (0.351) | |
| Coefficients | -1.092*** (0.314) | |
| Interaction | 0.356 (0.275) | |

*Note.* Standard errors in parentheses.

*** P < .01, ** P < .05, * P < .10.
